# Supplementary material for: Downregulation of macrophage Irs2 by hyperinsulinemia impairs IL-4-indeuced M2a-subtype macrophage activation in obesity
Source: Nat Commun. 2018 Nov 19;9:4863. doi: 10.1038/s41467-018-07358-9 (PMC6242852; doi:10.1038/s41467-018-07358-9)
Supplement: Supplementary file 1 — Supplementary file [file 41467_2018_7358_MOESM1_ESM.pdf]

Supplementary Fig.1

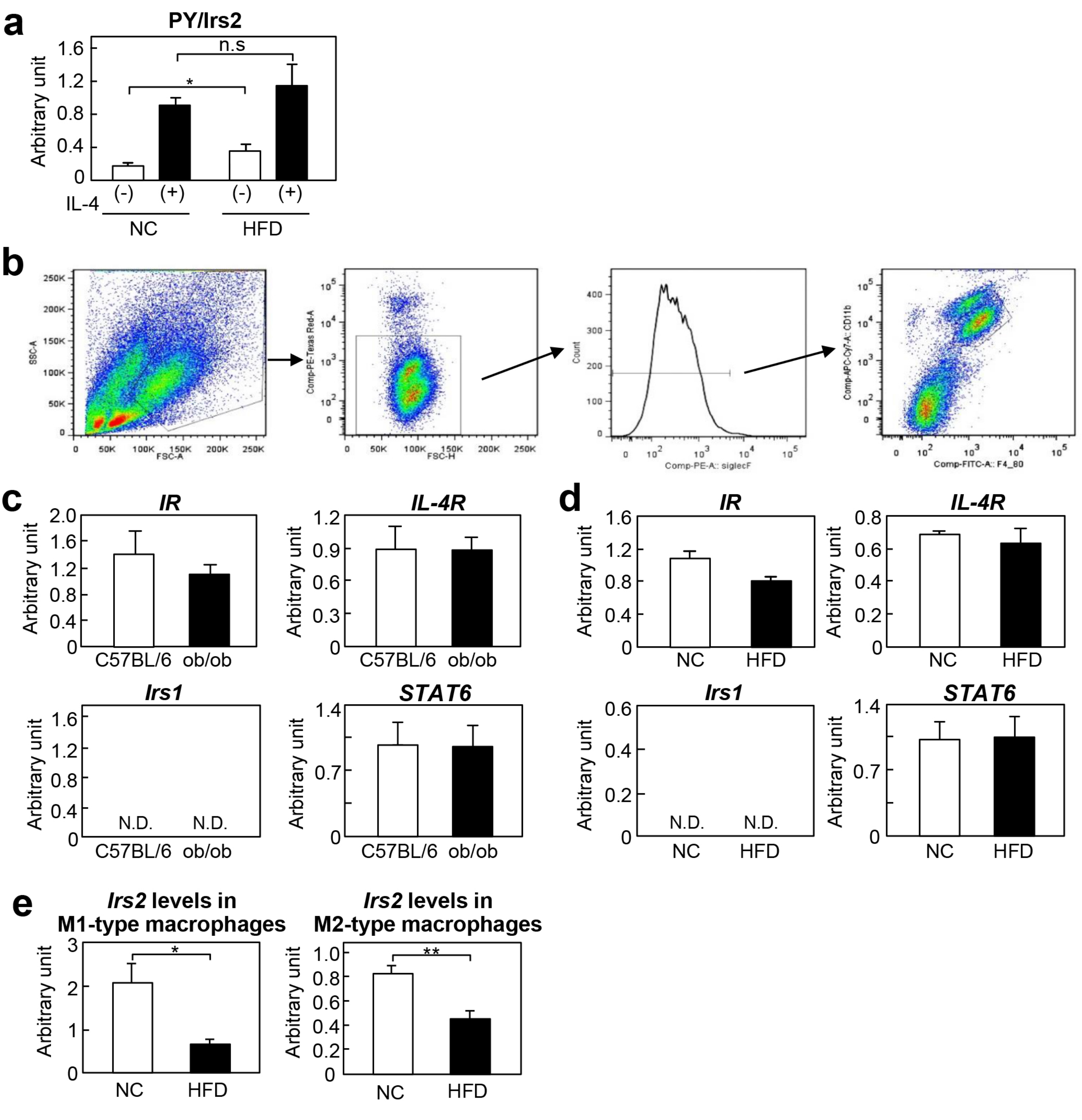

Expression levels in MΦs isolated from the SVF of the adipose tissue of the ob/ob and HF diet-fed mice.
(**a**) Phosphorylation/protein levels of Irs2 in peritoneal MΦs derived from NC and HF diet-fed mice (n = 4). (**b**) Isolation of siglecF$^-$CD11b$^+$F4/80$^+$cells from the SVF of the adipose tissue by flow cytometry. (**c, d**) Expression levels of *IR*, *IL-4R*, *Irs1* and *STAT6* mRNA in the siglecF$^-$CD11b$^+$F4/80$^+$ cells isolated from the SVF of the adipose tissue of the ob/ob and HF diet-fed mice (n = 4). (**e**) Expression levels of *Irs2* in M1-type and M2-type MΦs from NC and HF diet-fed mice (n = 3-7). N.D. is "not-detected". Data are mean±SEM. followed by one-way ANOVA with a *post-hoc* test or Student's *t* test. *, $P < 0.05$; **, $P < 0.01$.

Supplementary Fig.2

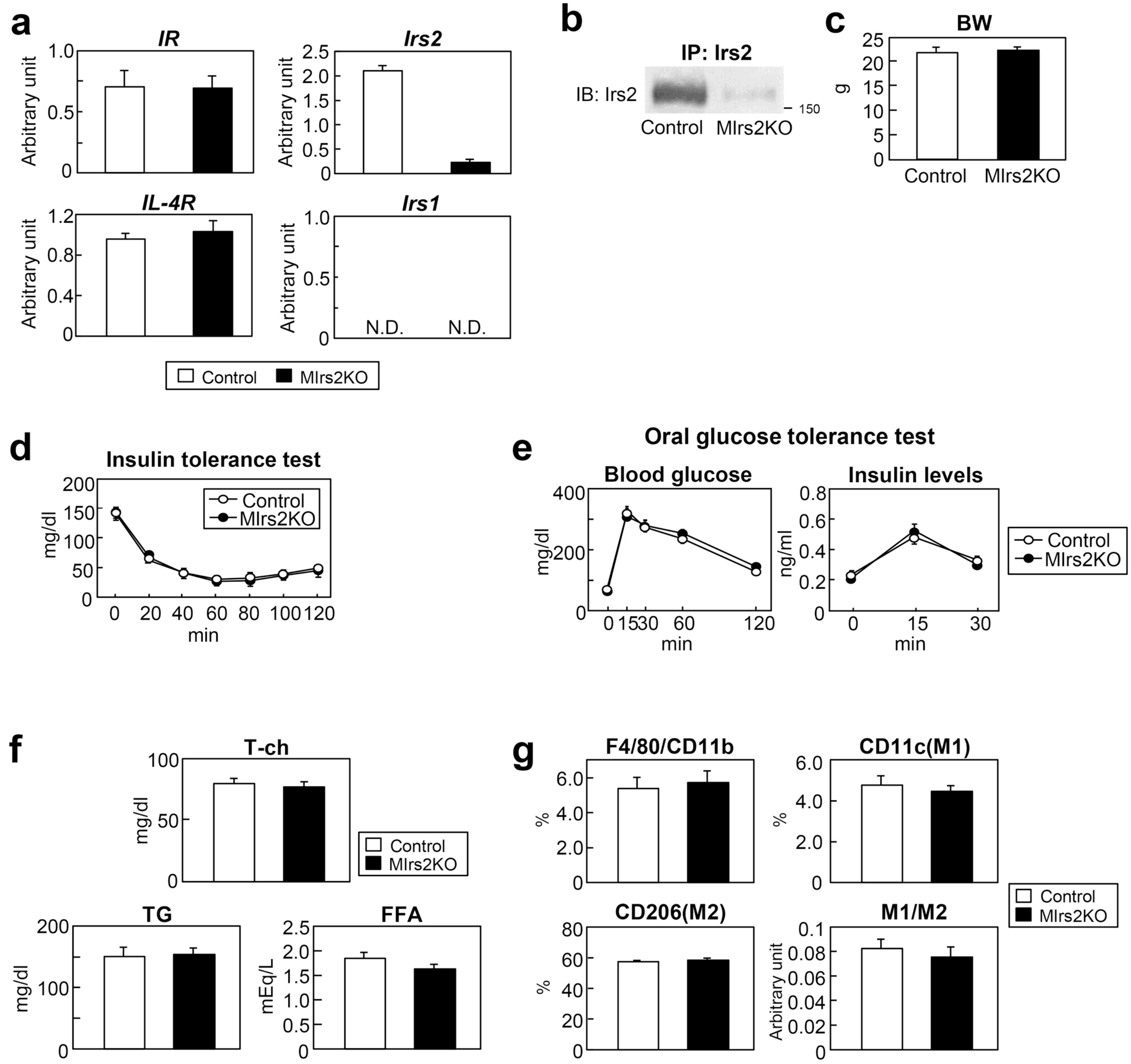

Phenotypes of M*Irs2*KO mice under the NC diet condition.

(**a**) mRNA expression levels of *IR*, *IL-4R*, *Irs1* and *Irs2* in the BMDM of the control and M*Irs2*KO mice (n = 6). (**b**) Irs2 protein levels in the BMDM of the control and M*Irs2*KO mice. (**c-f**) Body weight (BW), insulin tolerance test results (ITT), oral glucose tolerance test results (OGTT) and lipid profiles of the control and M*Irs2*KO mice under the normal chow (NC) diet condition (n = 6-9). (**g**) Percentages of siglecF-CD11b+F4/80+ cells, siglecF-CD11b+F4/80+CD11c+ cells and siglecF-CD11b+F4/80+CD206+ cells in the SVF of the adipose tissue derived from the NC diet-fed M*Irs2*KO mice (n = 4-6). N.D. is “not-detected”. Data are mean±SEM. followed by Student’s t test.

Supplementary Fig.3

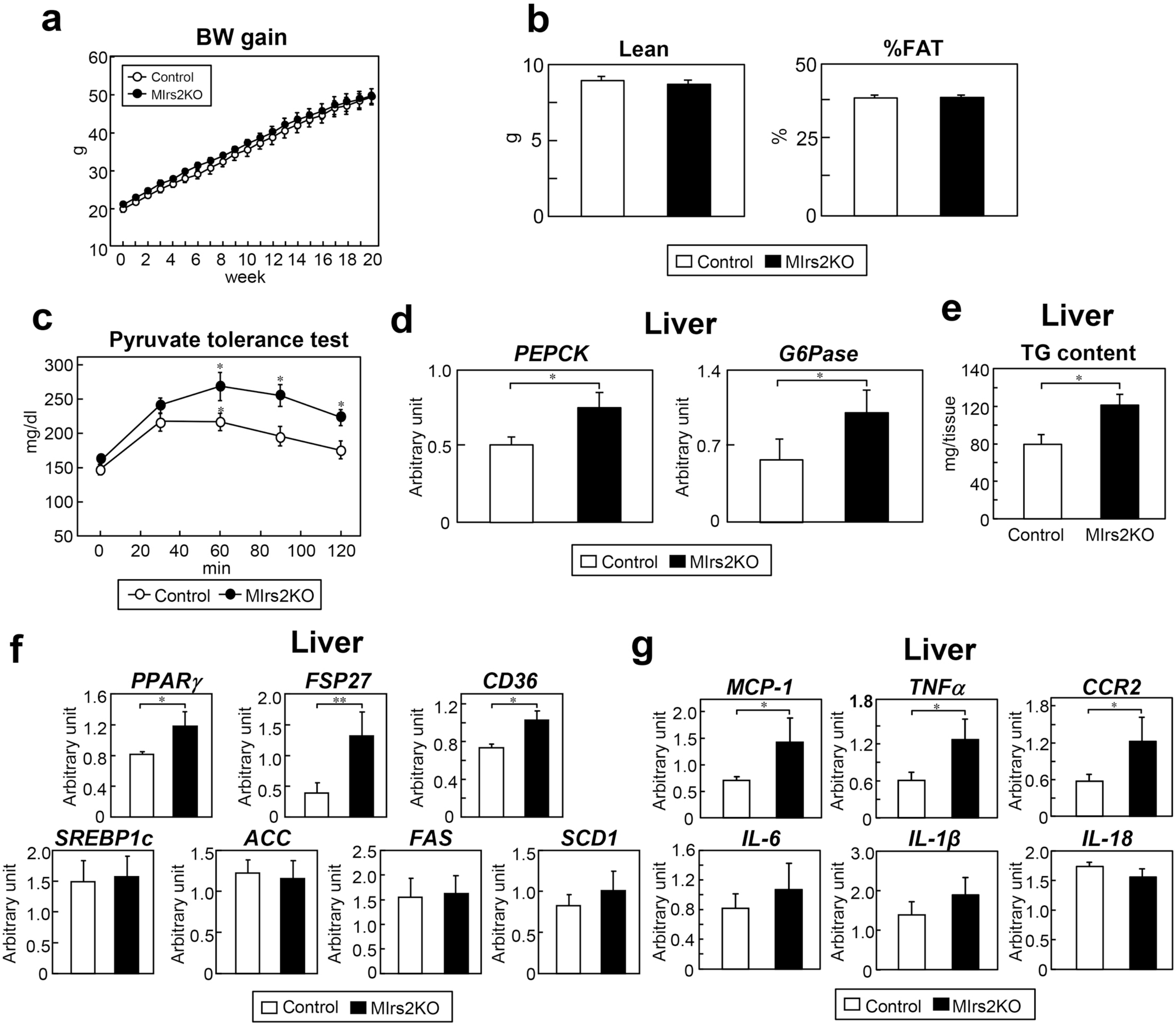

Gluconeogenesis and lipogenesis in the liver of M*Irs2*KO mice under the HF diet condition

(**a**, **b**) BW gain, lean body mass and percent body fat (%FAT) of the control and M*Irs2*KO mice reared on a HF diet (n = 8-9). (**c**) Pyruvate tolerance test in the M*Irs2*KO mice (n = 7-9). (**d**) Expression levels of gluconeogenesis-associated genes in the M*Irs2*KO mice under the HF diet condition (n = 9-10). (**e**) Hepatic TG content in the M*Irs2*KO mice (n = 5). (**f**) Expression levels of lipogenesis-associated genes in the M*Irs2*KO mice under the HF diet condition (n = 9-10). (**g**) Quantitative RT-PCR analysis of the genes encoding inflammatory cytokines in the livers of the M*Irs2*KO mice (n = 9-10). N.D. is “not-detected”. Data are mean±SEM. followed by Student’s t test. *, $P < 0.05$; **, $P < 0.01$.

Supplementary Fig.4

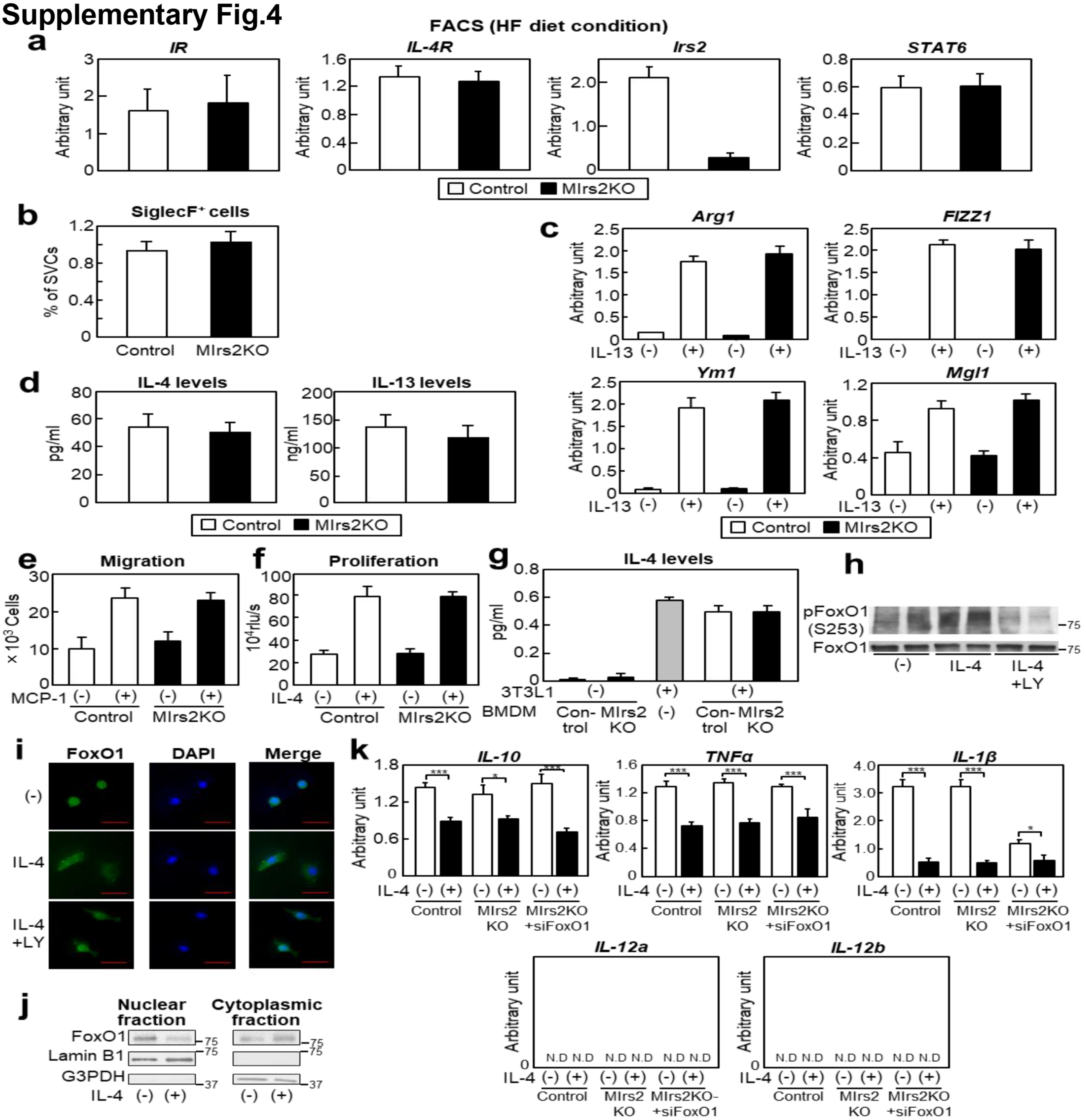

The role of FoxO1 in M2a-subtype MΦ after IL-4 stimulation.

(**a**) *IR*, *IL-4R*, *Irs2* and *STAT6* expression levels in the siglecF-CD11b+F4/80+ cells of M*Irs2*KO mice reared on a HF diet (n = 10-14). (**b**) The percentage of SiglecF+ cells in the SVCs of M*Irs2*KO mice fed a HF diet (n = 9-11). (**c**) Expression levels of the M2a-subtype MΦ marker genes in the BMDM of the control and M*Irs2*KO mice 48h after IL-13 stimulation (n = 3). (**d**) IL-4 and IL-13 levels in the WAT of the control and M*Irs2*KO mice (n = 5). (**e**) Migration of the BMDM of the control and M*Irs2*KO mice 48h after MCP-1 stimulation (n = 5-10). (**f**) Proliferation of the BMDM of the control and M*Irs2*KO mice 48h after IL-4 stimulation (n = 5-6). (**g**) IL-4 levels in the conditioned medium after co-culture of BMDM of M*Irs2*KO mice with or without 3T3-L1 cells for 24 h (n = 7-9). (**h**) FoxO1 phosphorylation and protein expression levels in the BMDM of the C57BL/6 mice after IL-4 stimulation with LY294002 treatment. (**i**) Immunohistochemical staining for FoxO1 in the BMDM of the C57BL/6 mice after IL-4 stimulation with LY294002 treatment (scale bar, 50 μm). (**j**) FoxO1 protein expression of nuclear and cytoplasmic fractions in the BMDM of the C57BL/6 mice after IL-4 stimulation. (**k**) Expression levels of *IL-10*, *TNFα*, *IL-1β*, *IL-12a*, and *IL-12b* in the siFoxO1-transfected BMDM of the M*Irs2*KO mice after IL-4 stimulation (n = 4-10). N.D. is "not-detected". Data are mean±SEM. followed by one-way ANOVA with a post-hoc test or Student's t test. *, $P < 0.05$; ***, $P < 0.001$.

Supplementary Fig.5

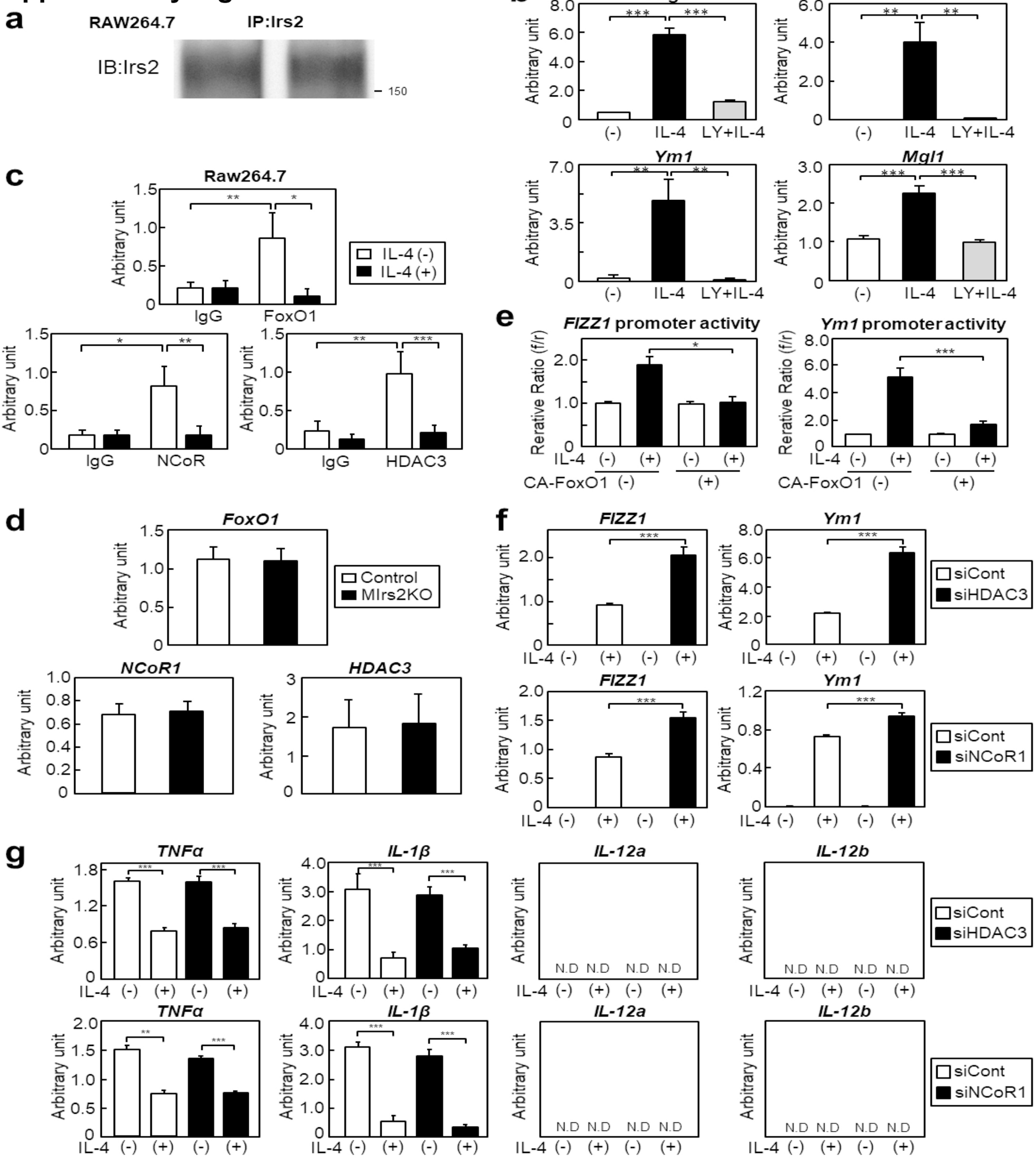

IL-4-induced *FIZZ1* and *Ym1* expressions were increased by the dissociation of the FoxO1/HDAC3/NCoR1 corepressor complex. (**a**) Irs2 protein expression in the RAW264.7 cells. (**b**) Expression levels of the M2a-subtype MΦ marker genes in the RAW264.7 cells after IL-4 stimulation with LY294002 treatment (n = 3). (**c**) Chip-qPCR using FoxO1, HDAC3 and NCoR1 antibody in the RAW264.7 cells before and after IL-4 stimulation (n = 5-10). (**d**) FoxO1, HDAC3 and NCoR1 expression levels in the ATMs of control and M*Irs2*KO mice (n = 12-15). (**e**) *FIZZ1* and *Ym1* promoter activities after IL-4 stimulation with or without CA-FoxO1 transfection (n = 4-5). (**f**) Expression levels of *FIZZ1* and *Ym1* in the BMDM of the C57BL/6 mice after IL-4 stimulation with siHDAC3 or siNCoR1 transfection (n = 3-8). (**g**) Expression levels of *TNFα*, *IL-1β*, *IL-12a*, and *IL-12b* in siHDAC3- or siNCoR1-transfected BMDM of the C57BL/6 mice after IL-4 stimulation (n = 3-8). N.D. is "not-detected". Data are mean±SEM. followed by one-way ANOVA with a post-hoc test or Student's t test. *, $P < 0.05$; **, $P < 0.01$; ***, $P < 0.001$.

Supplementary Fig.6

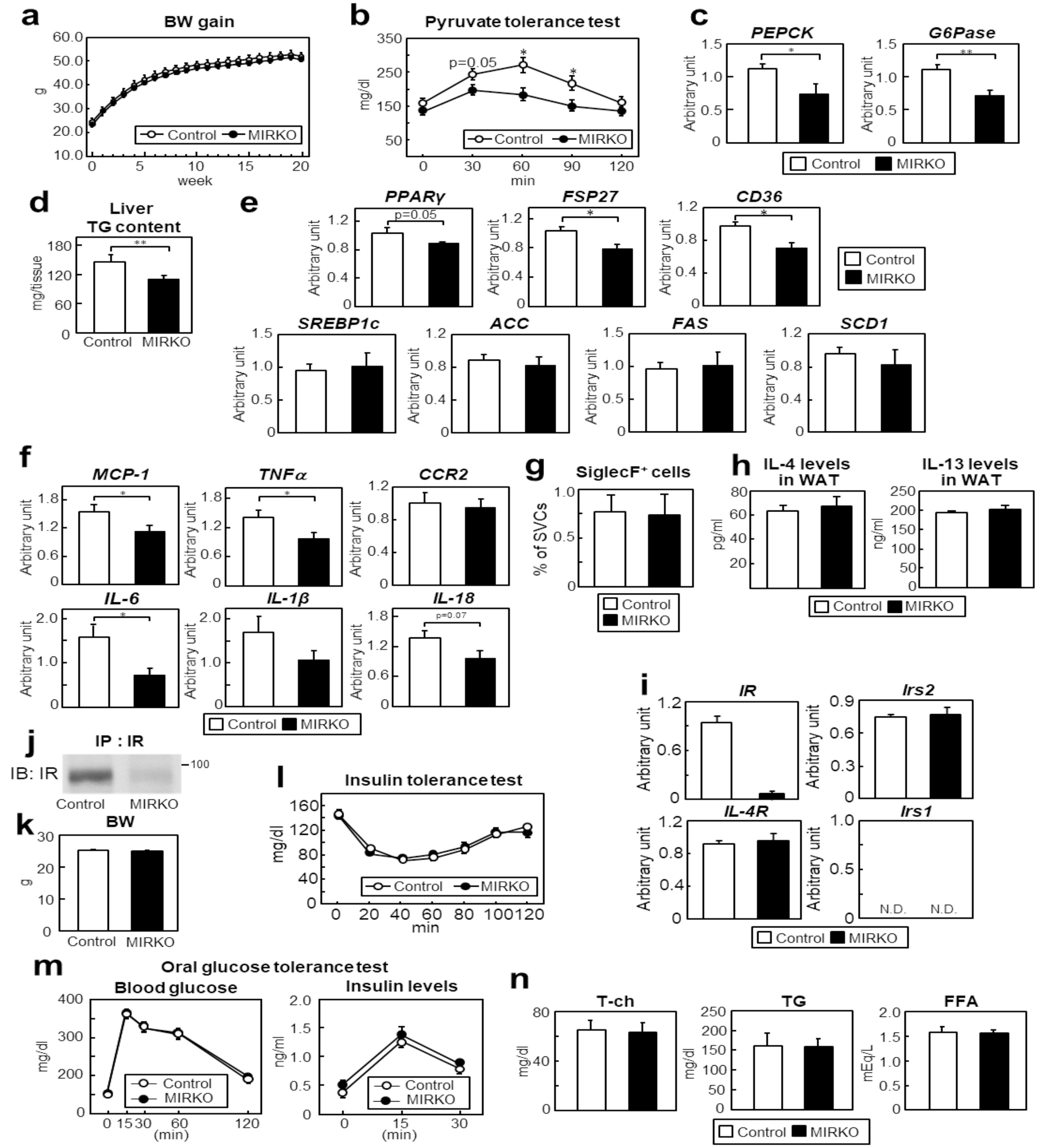

Phenotypes of M*IR*KO mice under the NC and HF diet condition.

(**a**) BW gain in the control and M*IR*KO mice reared on a HF diet (n = 9-10). (**b**) Pyruvate tolerance test in the M*IR*KO mice (n = 8). (**c**) Expression levels of gluconeogenesis-associated genes in the HF diet-fed M*IR*KO mice (n = 6-8). (**d**) Hepatic TG content in the M*IR*KO mice (n = 4-5). (**e**) Expression levels of lipogenesis-associated genes in the HF diet-fed M*IR*KO mice (n = 6-8). (**f**) Quantitative RT-PCR analysis of the genes encoding inflammatory cytokines in the livers of the M*IR*KO mice (n = 6-8). (**g**) The percentage of SiglecF+ cells in the SVCs of M*IR*KO mice fed a HF diet (n = 4-5). (**h**) IL-4 and IL-13 levels in the WAT of the control and M*IR*KO mice (n = 5). (**i**) mRNA expression levels of *IR*, *IL-4R*, *Irs1* and *Irs2* in the BMDM of the control and M*IR*KO mice (n = 9-10). (**j**) IR protein levels in the BMDM of the control and M*IR*KO mice. (**k-n**) Body weight (BW), insulin tolerance test (ITT) results, oral glucose tolerance test (OGTT) results and lipid profiles in the control and M*IR*KO mice under the NC diet condition (n = 6-10). N.D. is "not-detected". Data are mean±SEM. followed by Student's t test. *, $P < 0.05$; **, $P < 0.01$.

Supplementary Fig.7

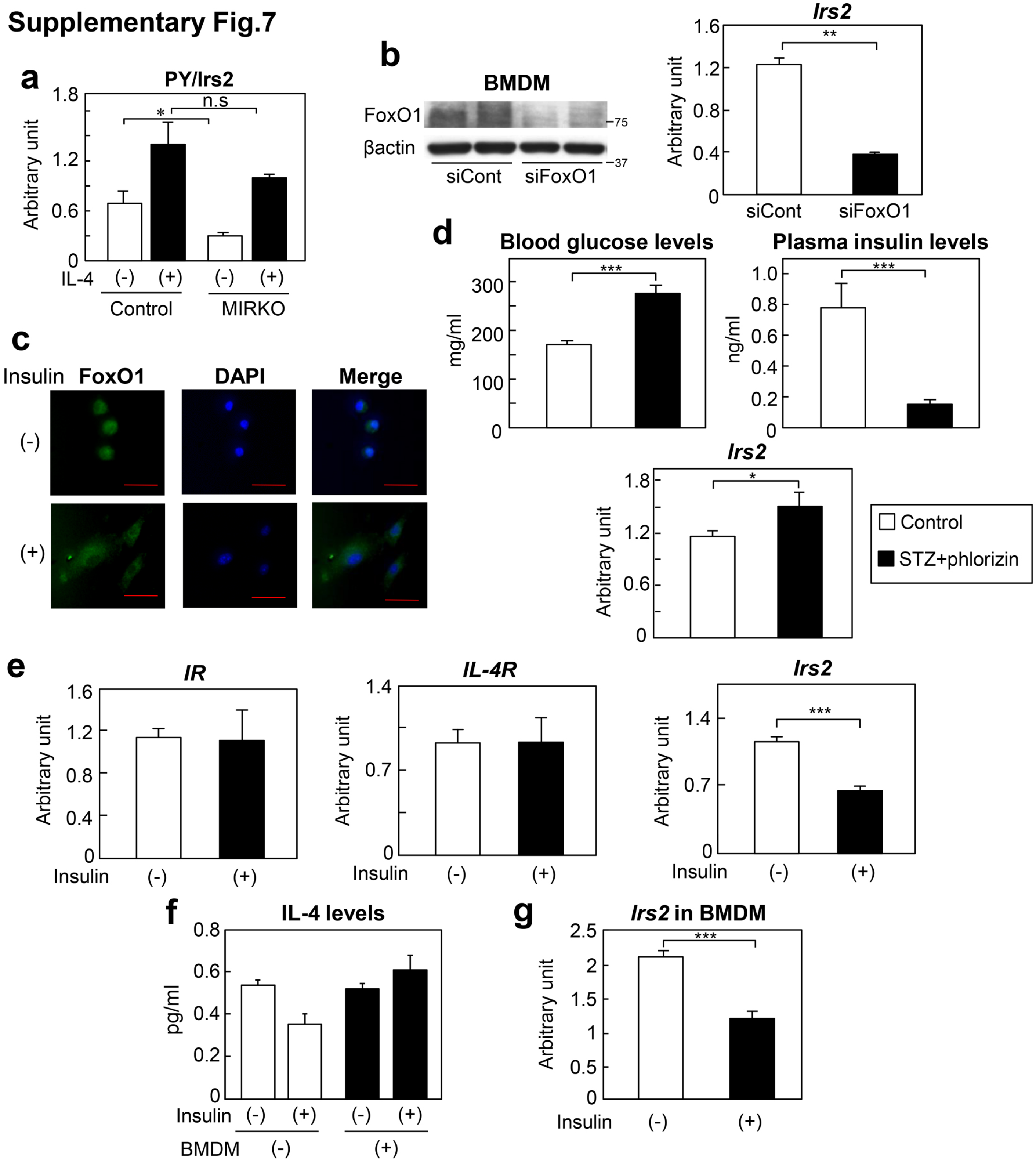

Expression levels of *Irs2* in MΦs after insulin stimulation.

(**a**) Phosphorylation/protein levels of Irs2 in the peritoneal MΦs of HF diet-fed M*IR*KO mice (n = 3-6). (**b**) *Irs2* expression levels in the BMDM after siFoxO1 transfection (n = 6). (**c**) Immunohistochemical staining for FoxO1 in the BMDM after insulin stimulation (scale bar, 50 μm). (**d**) Blood glucose levels, plasma insulin levels and expression levels of *Irs2* in the peritoneal MΦs in mice after STZ plus phlorizin treatment (n = 5-10). (**e**) Expression levels of *IR*, *IL-4R*, and *Irs2* in the BMDM 3 h after insulin stimulation (n = 8-10). (**f**) IL-4 levels in the conditioned medium after co-culture of BMDM and 3T3-L1 cells after insulin stimulation for 24 h (n = 6). (**g**) *Irs2* expression levels in BMDM co-cultured with 3T3-L1 cells after insulin stimulation for 24 h (n = 11-20). Data are mean±SEM. followed by one-way ANOVA with a post-hoc test or Student's t test. *, $P < 0.05$; **, $P < 0.01$; ***, $P < 0.001$.

Supplementary Fig.8

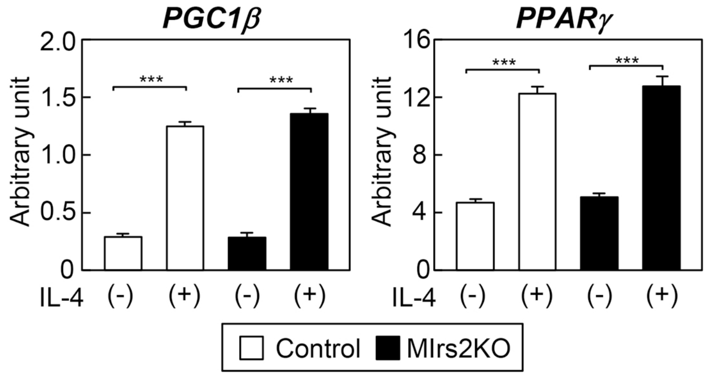

PGC1β and *PPARγ* expression levels did not differ between the control and M*Irs2*KO mice after IL-4 treatment.
Expression levels of *PGC1β* and *PPARγ* in the BMDM of the control and M*Irs2*KO mice after IL-4 stimulation (n = 6-8). Data are mean±SEM. followed by Student's t test. ***, $P < 0.001$.

Supplementary Fig.9

a

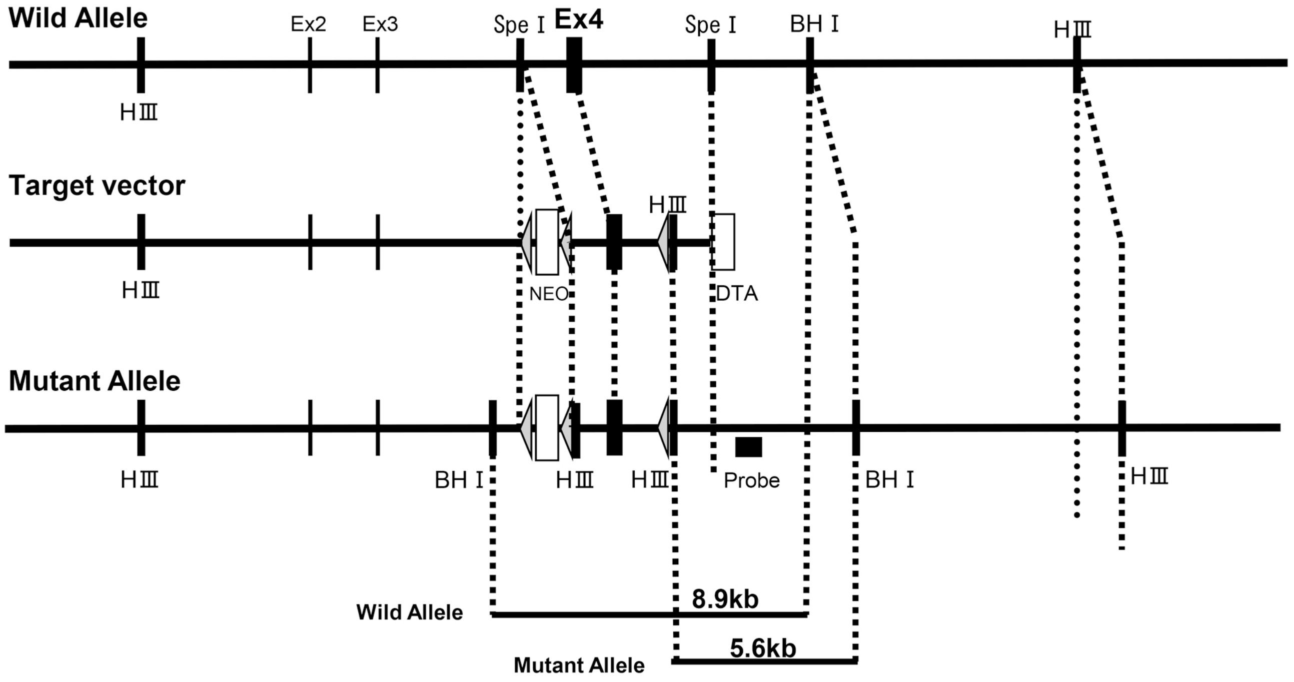

b

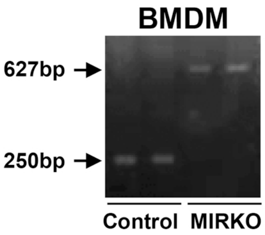

Generation of M*IR*KO mice
(**a**) Targeting strategy to insert LoxP sites into the *IR* gene. (**b**) PCR analysis of genomic DNA in MΦs to detect Cre-mediated recombination in the M*IR*KO mice.

Supplementary Fig.10

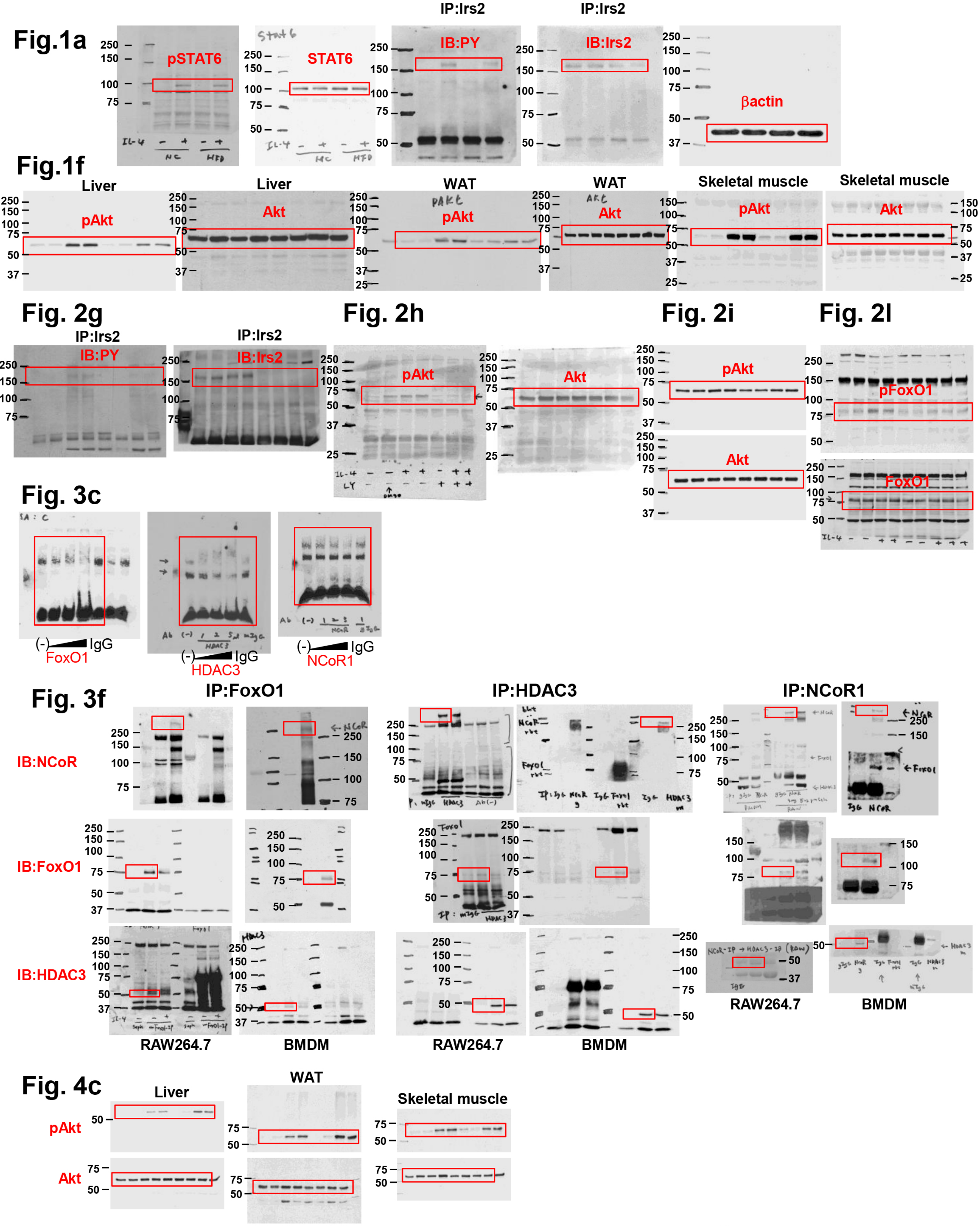

Uncropped images of immunoblot data.

Supplementary Fig.11

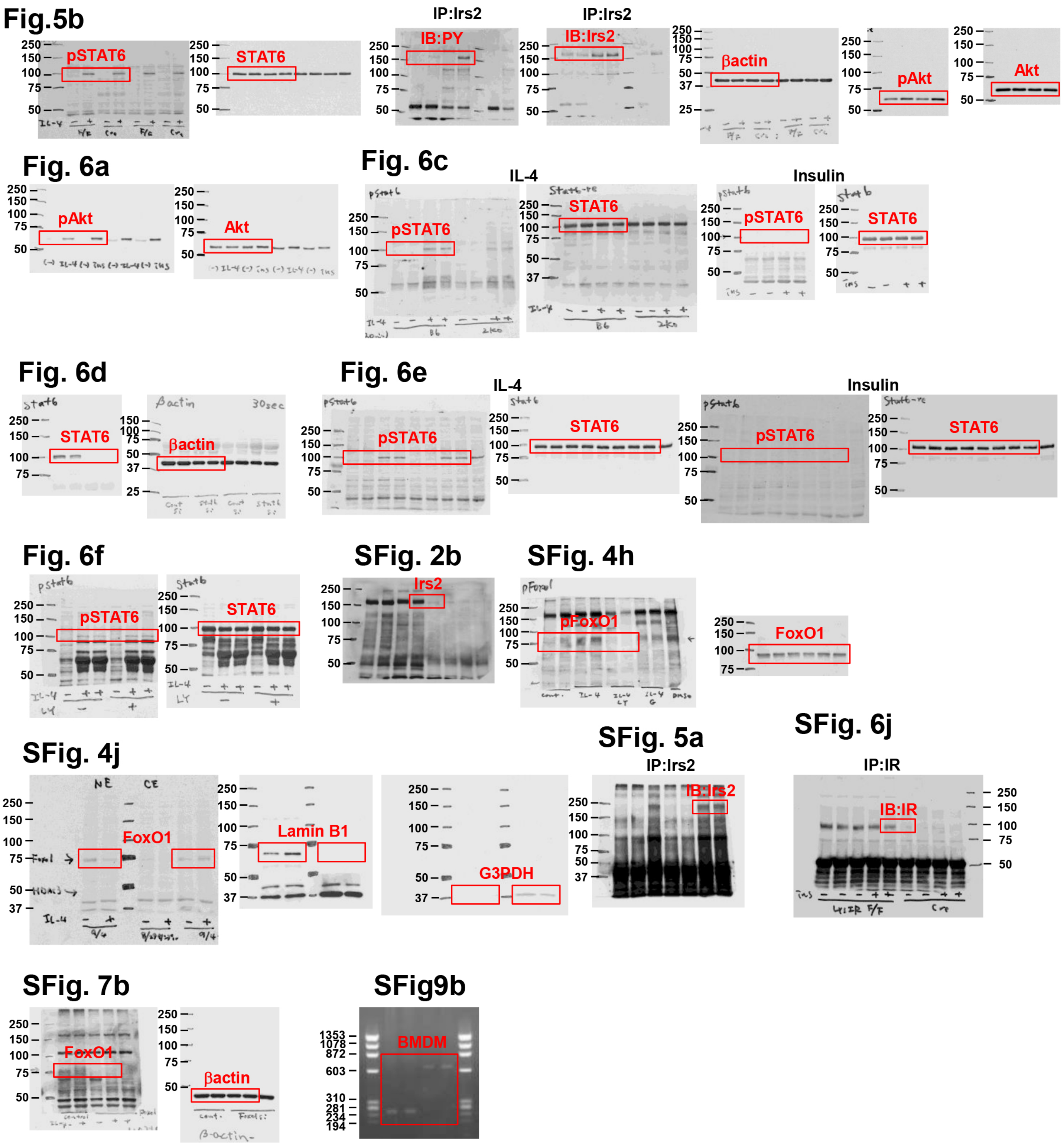

Uncropped images of immunoblot and PCR data.
